# Pre-PRIS? Prospective monitoring for early markers of propofol infusion syndrome

**DOI:** 10.1186/cc12329

**Published:** 2013-03-19

**Authors:** M Stovell, S Smith, M Udberg, W Loh, P Nair

**Affiliations:** 1 Walton Centre Neurology & Neurosurgery, Liverpool, UK

## Introduction

The use of propofol for sedation in intensive care has been associated with the propofol infusion syndrome (PRIS) characterised by cardiac dysfunction, metabolic acidosis, renal failure, rhabdomyolysis and hyperlipidaemia. We prospectively monitor biochemical markers that we believe demonstrate early signs of this dangerous, often fatal syndrome. When this Pre-PRIS state is identified, propofol is withdrawn whilst the syndrome is still reversible.

## Methods

We prospectively audited our monitoring of these markers over a 4-month period in propofol-sedated patients: propofol infusion rate, creatine kinase (CK), triglycerides (TG), creatinine, lactate, pH and base deficit. We defined the criteria for Pre-PRIS as requiring a CK ≥320 mmol/l that had doubled from its base level and a rise in TG ≥1.7 IU/l; both that followed a trend with propofol dose.

## Results

Data were collected from 54 adults; 50 received an infusion for ≥24 hours. We noted a significant CK rise in 11 (22%) of our patients that could be attributed to propofol alone. They all had raised TG (≥1.7 IU/l); six (12%) were markedly raised (≥3.4 IU/l). No patients became significantly acidotic, developed cardiac arrhythmias or renal failure as a result of propofol. In the 11 patients who met our criteria, highest daily propofol dose varied from 1.5 to 4 mg/kg/hour. Pre-PRIS developed from between 2 and 6 days of infusion. The mean propofol dose in patients displaying Pre-PRIS was 2.2 mg/kg/hour whilst in those not meeting our criteria it was 2.0 mg/kg/hour. No patients developed sequelae of full PRIS. See Table 1.

**Table 1:** 

	Total	Trauma	Nontrauma
≥24 hours infusion	50	34	16
↑CK + ↑TG ≥3.4	6	6	0
↑CK + ↑TG ≥1.7	5	3	2

## Conclusion

We propose that a paired rise in CK and TG that can be attributed to propofol alone represents a Pre-PRIS state that is at risk of developing into full PRIS. We noted this in 22% of our patients, all on modest doses of propofol. It is unclear what proportion of patients will develop the full syndrome as it is not ethically possible to continue propofol in this situation. We advocate daily monitoring of CK and TG to identify Pre-PRIS so that propofol can be reduced or substituted to avoid the morbidity and mortality of the full syndrome.

